# Di­methyl­ammonium 2,4,5-tri­carb­oxy­benzoate: an example of the deca­rbonylation of *N*,*N*-di­methyl­formamide in the presence of a metal and a benzene­polycarb­oxy­lic acid. Is zirconium(IV) the *Tsotsi*?

**DOI:** 10.1107/S2056989016014948

**Published:** 2016-10-04

**Authors:** S. T. Hulushe, E. C. Hosten, G. M. Watkins

**Affiliations:** a Department of Chemistry, Rhodes University, PO Box 94, Grahamstown, South Africa; b Department of Chemistry, Nelson Mandela University, Summerstrand, PO Box 77000, South Africa

**Keywords:** crystal structure, deca­rbonylation, multiple hydrogen bonding, 1,2,4,5-benzene­tetra­carb­oxy­lic acid, *Tsotsi*

## Abstract

The title salt, C_2_H_8_N^+^·C_10_H_5_O_8_^−^, was the unexpected product of an attempt to prepare a Zr^IV^ metal–organic framework with benzene-1,2,4,5-tetra­carb­oxy­lic acid (1,2,4,5-H_3_B4C). In the reaction, the DMF solvent has been decarb­on­yl­ated, forming the di­methyl­ammonium cation, with one proton lost from the tetra­carb­oxy­lic acid. It is proposed that the Zr^IV^ salt acts as a *Tsotsi* or robber, plundering CO from the DMF mol­ecule. The resulting salt crystallizes with two cations and two anions in the asymmetric unit. An intra­molecular hydrogen bond forms between a carb­oxy­lic acid substituent and the carboxyl­ate group of each of the monodeprotonated (1,2,4,5-H_3_B4C^−^) anions. In the crystal, an extensive array of O—H⋯O, N—H⋯O and C—H⋯O hydrogen bonds generates a three-dimensional network, with columns of cations and anions forming along the *b* axis.

## Abstract 2

The paper reports the mol­ecular and crystal structure of the salt (CH_3_)_2_NH_2_^+^·C_10_H_5_O_8_^−^, with the cation formed by the deca­rbonylation of DMF solvent.

## Chemical context   

The term *Tsotsi* is South African township slang for a street gangster or hoodlum who is known to mug unsuspecting passers-by and steal their goods. Identifying a possible *Tsotsi* on the street is part of everyday township life. We attempted to grow a single crystal of a zirconium-based metal–organic framework incorporating 1,2,4,5-benzene­tetra­carb­oxy­lic acid that we had previously synthesized in powder form, in a di­methyl­formamide, DMF, solution. Instead this yielded crystals of the unanti­cipated title compound (I). The unexpected deca­rbonylation of *N*,*N*-di­methyl­formamide (DMF) has led us to ponder the possible characteristics of the reagents used that led to this ‘plundering’ of the DMF. De­carbonyl­ation of DMF has previously been shown to occur under slow evaporation conditions in the presence of coordination complexes (Siddiqui *et al.*, 2012 ▸; Chen *et al.*, 2007 ▸; Karpova *et al.*, 2004 ▸). In these reports, the nitrate salts of Mg^II^ (Siddiqui *et al.*, 2012 ▸), Pb^II^ (Chen *et al.*, 2007 ▸), Ho^III^ and the chloride salt of Nd^III^ (Karpova *et al.*, 2004 ▸) ions were suggested to play a unique catalytic role in the observed deca­rbonylation reaction. The form of the metals in these reactions was thought to be as six-coordinate metal complexes This suggests that, in the decarb­onylation reaction observed here, the active decarb­on­ylation agent could be the chloride salt of Zr^IV^ as this is also likely to be six-coordinate in solution.
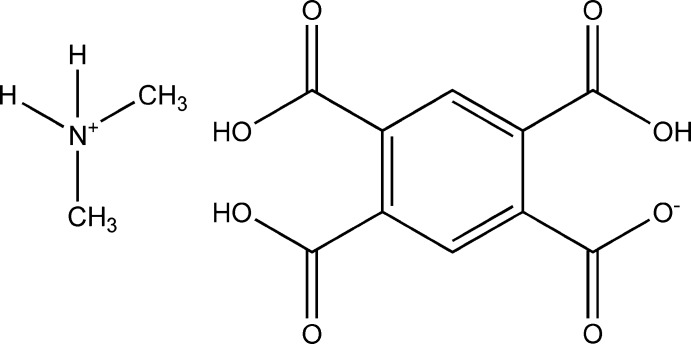


The other potential deca­rbonylation catalyst in this reaction is the benzene­tetra­carb­oxy­lic acid. However, Dale and coworkers have studied the slow evaporation reactions of 1,4-benzene­dicarb­oxy­lic acid (terephthalic acid; 1,4-H_2_B2C), 1,2,3-benzene­tri­carb­oxy­lic acid (hemimellitic acid; 1,2,3-H_3_B3C) and 1,2,4,5-benzene­tetra­carb­oxy­lic acid (pyromellitic acid; 1,2,4,5-H_4_B4C) in the absence of metal complexes and no deca­rbonylation of DMF was observed (Dale & Elsegood, 2004 ▸).

**Figure 1:**
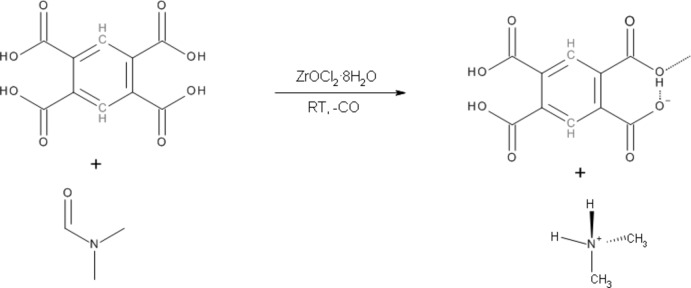
The reaction procedure used in the preparation of (I).

Clearly this further implicates the zirconium(IV) as the *Tsotsi* in this deca­rbonylation reaction, stealing CO from the DMF and forming the di­methyl­ammonium cation (Fig. 1 ▸). While the detailed mechanism of the deca­rbonylation process remains unclear, it is most likely that the formation of this salt is initiated by the zirconium(IV) *Tsotsi*.

## Structural commentary   

**Figure 2:**
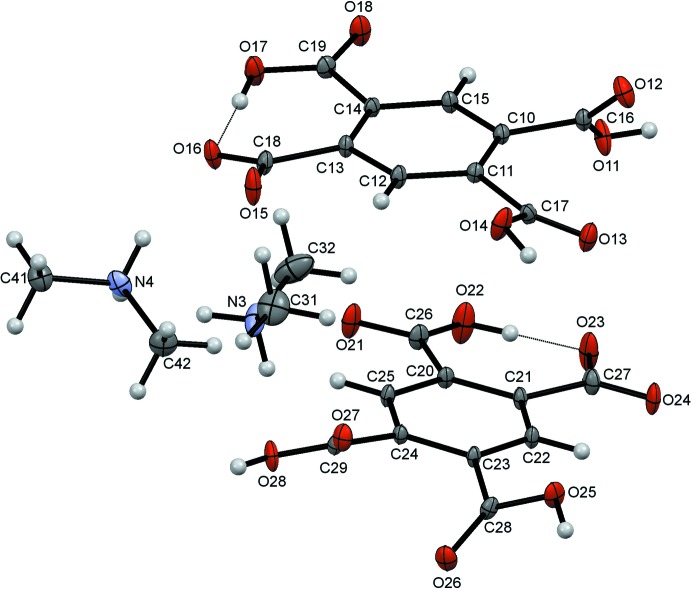
The asymmetric unit of (I), showing the atom-numbering scheme. Displacement ellipsoids are drawn at the 50% probability level. Intra­molecular hydrogen bonds are drawn as dashed lines.

**Figure 3:**
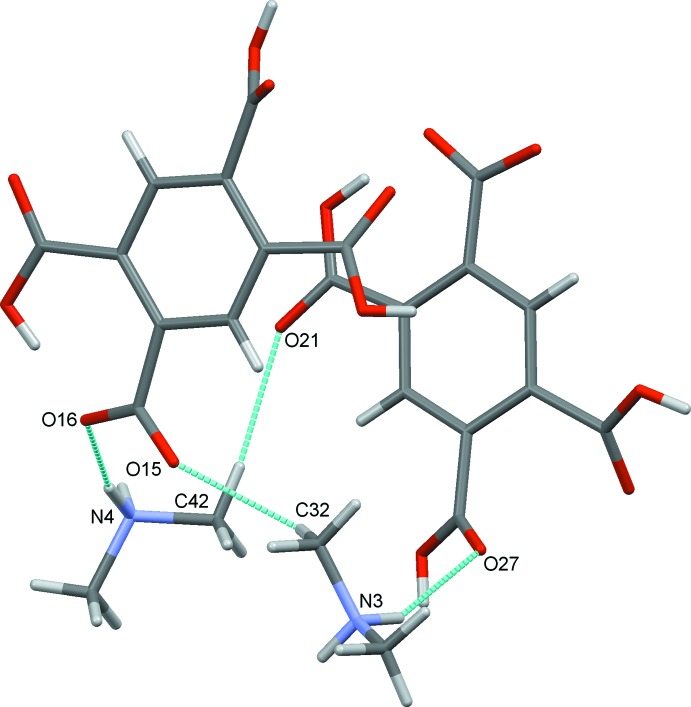
The asymmetric unit of (I), showing the hydrogen bonds formed between the cations and anions. In this and subsequent figures, hydrogen bonds are shown as blue dashed lines.

The asymmetric unit of the title salt C_2_H_8_N^+^ C_10_H_5_O_8_^−^, (I), consists of two anions, 1 and 2 and two cations, 3 and 4, differentiated by the leading numbers in the numbering scheme, Fig. 2 ▸. Within the asymmetric unit, both cations and anions are linked by strong N—H⋯O and weaker C—H⋯O hydrogen bonds, Table 1 ▸, Fig. 3 ▸. Bond distances and angles in the approximately tetra­hedral di­methyl­ammonium cations are unremarkable.

The benzene rings of the anions are inclined to one another by 6.56 (3)°. In both anions, the two carboxyl­ate substituents lie reasonably close to the benzene ring planes, inclined at 16.16 (19)° for 1 and 6.01 (5)° for 2. One carb­oxy­lic acid substituent in each cation also lies close to these planes, [5.85 (8)° for 1 and 6.25 (9)° for 2]. This planarity is doubtless aided by the two intra­molecular O17—H17*A*⋯O16 and O22—H22*A*⋯O23 hydrogen bonds that form between a carboxyl­ate oxygen and the OH group of an adjacent carb­oxy­lic acid substituent in each of the discrete anions, Fig. 2 ▸. Each encloses an *S*7 ring. The other two carb­oxy­lic acid substituents in both anions lie well out of the benzene ring planes with dihedral angles ranging from 75.4 (4) to 37.23 (15)°.

## Supra­molecular features   

**Figure 4:**
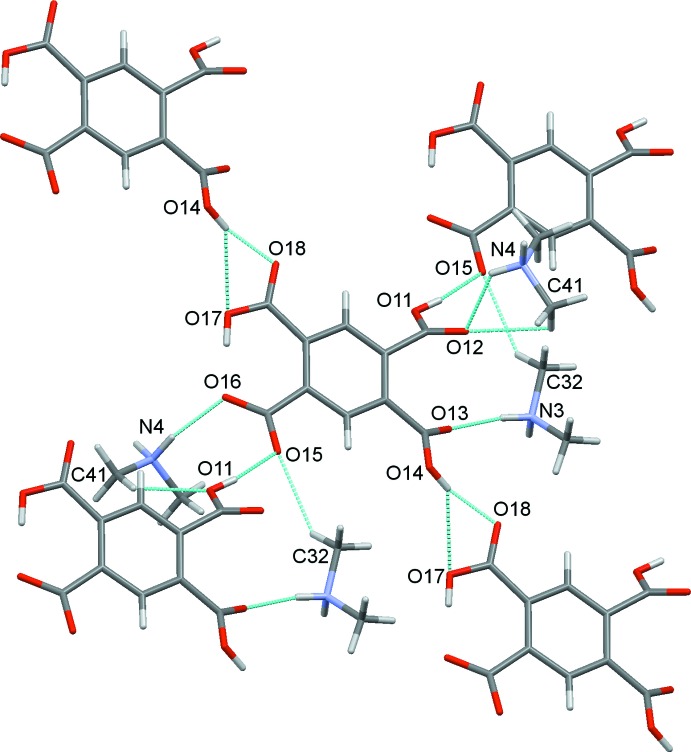
The immediate environment of anion 1.

**Figure 5:**
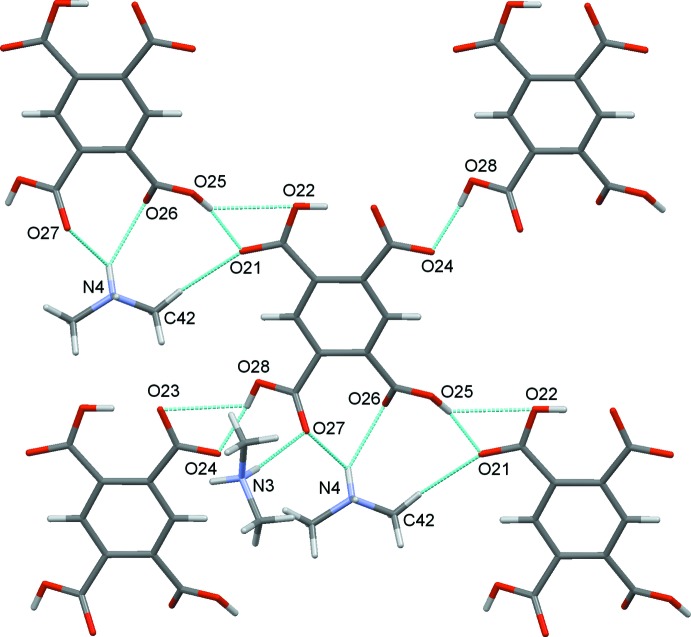
The immediate environment of anion 2.

In the crystal structure, a myriad of classical O—H⋯O and N—H⋯O hydrogen bonds are found together with non-classical C—H⋯O hydrogen bonds. These are detailed in Table 1 ▸. Each individual anion of type 1 binds to four other type 1 anions through O—H⋯O hydrogen bonds. Each also binds to four cations, two of type 3 and two of type 4, through N—H⋯O and C—H⋯O hydrogen bonds, Fig. 4 ▸. Similarly, each type 2 anion binds to four other discrete type 2 anions and to three cations one of type 3 and two of type 4, Fig. 5 ▸.

**Figure 6:**
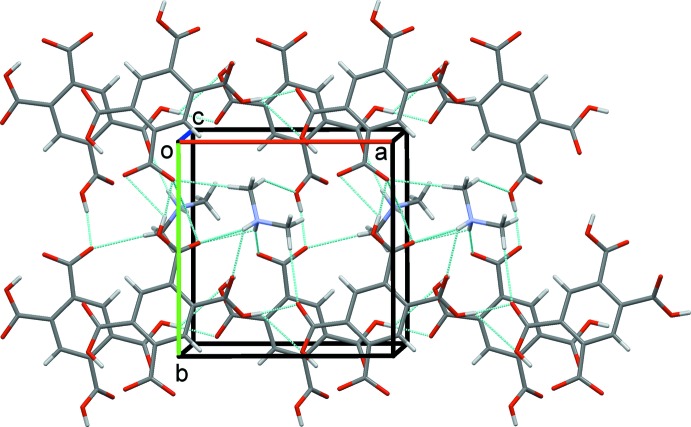
Sheets formed in the *ab* plane by the cations and anions of (I).

**Figure 7:**
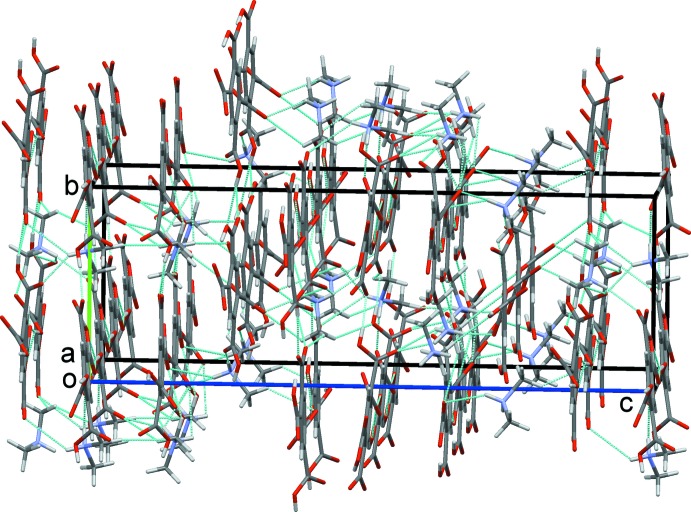
Overall packing for (I), viewed along the *a*-axis direction.

Layers built from alternating rows of cations and anions form in the *ab* plane, Fig. 6 ▸. These layers are further linked by N—H⋯O and C—H⋯O contacts to form a three-dimensional network comprised of linked columns of cations and anions, Fig. 7 ▸.

## Database survey   

A search of the Cambridge Structural Database (Version 5.37, update November 2015; Groom *et al.*, 2016 ▸) for 1,2,4,5-H_3_B4C^−^ anion yielded 46 hits and of these 35 are purely organic compounds. One particular compound, YIRFOV, reports a 1,2,4,5-H_3_B4C^−^ salt with a tetra­methyl­ammonium cation (Cunha-Silva *et al.*, 2008 ▸). This is very similar to the structure reported here. The principal difference between these structures is that the asymmetric unit of YIRFOV comprises one tetra­methyl­ammonium cation, one 1,2,4,5-H_3_B4C^−^ anion co-crystallized with half a fully protonated 1,2,4,5-H_4_B4C mol­ecule that lies on a centre of inversion. In YIRFOV, the crystal packing is also mediated by an extensive hydrogen-bonding network.

## Synthesis and crystallization   

A 2 mL aqueous solution of ZrOCl_2_·8H_2_O (0.04 g, 0.124 mmol) was suspended in 0.5 mL *N*,*N*-di­methyl­formamide (DMF). A 2 mL aqueous solution of 1,2,4,5-H_4_B4C 0.032 g, 0.124 mmol) was similarly suspended in 0.5 mL DMF and the two solutions were combined in a small sample vial. This was placed inside a larger sample vial. 0.5 mL of deionized water was added before it was covered and left until crystallization was complete. After three weeks, yellow–brown cubic crystals formed. These were isolated and used for the X-ray crystallographic analysis.

## Refinement   

Crystal data, data collection and structure refinement details are summarized in Table 2 ▸. All non-hydrogen atoms were refined anisotropically. Carbon-bound hydrogen atoms were placed in calculated positions and were included in the refinement in the riding-model approximation, with *U*_iso_(H) set to 1.2*U*_eq_(C). The hydrogen atoms of the methyl groups were allowed to rotate with a fixed angle around the C—C bond to best fit the experimental electron density, with *U*_iso_(H) = 1.5*U*_eq_(C). The H atoms of the hydroxyl groups were allowed to rotate with a fixed angle around the C—-O bond to best fit the experimental electron density with *U*_iso_(H) set to 1.5*U*_eq_(O).

## Supplementary Material

Crystal structure: contains datablock(s) I. DOI: 10.1107/S2056989016014948/sj5505sup1.cif

Structure factors: contains datablock(s) I. DOI: 10.1107/S2056989016014948/sj5505Isup2.hkl

Supporting information file. DOI: 10.1107/S2056989016014948/sj5505Isup3.cml

CCDC references: 1492232, 1492232

Additional supporting information: crystallographic information; 3D view; checkCIF report

## Figures and Tables

**Table 1:** Hydrogen-bond geometry (Å, °)

*D*—H⋯*A*	*D*—H	H⋯*A*	*D*⋯*A*	*D*—H⋯*A*
N3—H3*A*⋯O13^i^	0.91	1.86	2.762 (4)	169
N3—H3*B*⋯O27	0.91	1.99	2.798 (5)	147
N4—H4*A*⋯O26^ii^	0.91	2.32	3.025 (4)	134
N4—H4*A*⋯O27^ii^	0.91	2.49	2.918 (4)	110
N4—H4*A*⋯O12^iii^	0.91	2.19	2.830 (5)	127
N4—H4*B*⋯O16	0.91	1.99	2.879 (5)	167
O11—H11*A*⋯O15^iv^	0.84	1.73	2.560 (4)	171
O14—H14*A*⋯O17^v^	0.84	2.55	3.119 (4)	126
O14—H14*A*⋯O18^v^	0.84	1.77	2.583 (4)	161
O17—H17*A*⋯O16	0.84	1.57	2.409 (4)	176
O22—H22*A*⋯O23	0.88	1.49	2.370 (4)	179
O25—H25*A*⋯O21^v^	0.84	1.75	2.572 (4)	164
O25—H25*A*⋯O22^v^	0.84	2.59	3.181 (4)	129
O28—H28⋯O24^i^	0.84	1.74	2.571 (3)	168
C32—H32*C*⋯O15	0.98	2.54	3.234 (6)	128
C41—H41*C*⋯O11^i^	0.98	2.41	3.235 (5)	142
C42—H42*C*⋯O21	0.98	2.57	3.519 (6)	164

Symmetry codes: (i) 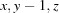; (ii) 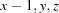; (iii) 
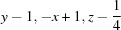
; (iv) 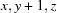; (v) 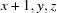.

**Table 2:** Experimental details

Crystal data	
Chemical formula	C_2_H_8_N^+^·C_10_H_5_O_8_^−^
*M*_r_	299.23
Crystal system, space group	Tetragonal, *P*4_1_
Temperature (K)	200
*a*, *c* (Å)	9.6621 (5), 27.8940 (17)
*V* (Å^3^)	2604.1 (3)
*Z*	8
Radiation type	Mo *K*α
μ (mm^−1^)	0.13
Crystal size (mm)	0.42 × 0.32 × 0.20

Data collection	
Diffractometer	Bruker APEXII CCD
Absorption correction	Numerical (*SADABS*; Bruker, 2010 ▸)
*T*_min_, *T*_max_	0.946, 1.000
No. of measured, independent and observed [*I* > 2σ(*I*)] reflections	47565, 6473, 6118
*R*_int_	0.025
(sin θ/λ)_max_ (Å^−1^)	0.667

Refinement	
*R*[*F* ^2^ > 2σ(*F* ^2^)], *wR*(*F* ^2^), *S*	0.046, 0.134, 1.15
No. of reflections	6473
No. of parameters	388
No. of restraints	2
H-atom treatment	H-atom parameters constrained
Δρ_max_, Δρ_min_ (e Å^−3^)	0.40, −0.27
Absolute structure	Flack *x* determined using 2780 quotients [(*I* ^+^)−(*I* ^−^)]/[(*I* ^+^)+(*I* ^−^)] (Parsons *et al.*, 2013 ▸)
Absolute structure parameter	−0.14 (15)

Computer programs: *APEX2* and *SAINT* (Bruker, 2010 ▸), *SHELXT* (Sheldrick, 2015*a* ▸), *SHELXL2014* (Sheldrick, 2015*b* ▸), *ShelXle* (Hübschle *et al.*, 2011 ▸), *ORTEP-3 for Windows* (Farrugia, 2012 ▸), *Mercury* (Macrae *et al.*, 2008 ▸) and *PLATON* (Spek, 2009 ▸).

## supplementary crystallographic information

### Crystal data

**Table:**

C_2_H_8_N^+^·C_10_H_5_O_8_^−^	*D*_x_ = 1.526 Mg m^−^^3^
*M**_r_* = 299.23	Mo *K*α radiation, λ = 0.71073 Å
Tetragonal, *P*4_1_	Cell parameters from 9896 reflections
*a* = 9.6621 (5) Å	θ = 2.2–28.3°
*c* = 27.8940 (17) Å	µ = 0.13 mm^−^^1^
*V* = 2604.1 (3) Å^3^	*T* = 200 K
*Z* = 8	Block, pale yellow
*F*(000) = 1248	0.42 × 0.32 × 0.20 mm

### Data collection

**Table:**

Bruker APEXII CCD diffractometer	6473 independent reflections
Radiation source: sealed tube	6118 reflections with *I* > 2σ(*I*)
Graphite monochromator	*R*_int_ = 0.025
Detector resolution: 8.3333 pixels mm^-1^	θ_max_ = 28.3°, θ_min_ = 2.1°
φ and ω scans	*h* = −12→12
Absorption correction: numerical (SADABS; Bruker, 2010)	*k* = −12→12
*T*_min_ = 0.946, *T*_max_ = 1.000	*l* = −37→37
47565 measured reflections	

### Refinement

**Table:**

Refinement on *F*^2^	Hydrogen site location: mixed
Least-squares matrix: full	H-atom parameters constrained
*R*[*F*^2^ > 2σ(*F*^2^)] = 0.046	*w* = 1/[σ^2^(*F*_o_^2^) + (0.0614*P*)^2^ + 1.8024*P*] where *P* = (*F*_o_^2^ + 2*F*_c_^2^)/3
*wR*(*F*^2^) = 0.134	(Δ/σ)_max_ < 0.001
*S* = 1.15	Δρ_max_ = 0.40 e Å^−^^3^
6473 reflections	Δρ_min_ = −0.27 e Å^−^^3^
388 parameters	Absolute structure: Flack *x* determined using 2780 quotients [(*I*^+^)-(*I*^-^)]/[(*I*^+^)+(*I*^-^)] (Parsons *et al.*, 2013)
2 restraints	Absolute structure parameter: −0.14 (15)

### Special details

**Table:**

Geometry. All esds (except the esd in the dihedral angle between two l.s. planes) are estimated using the full covariance matrix. The cell esds are taken into account individually in the estimation of esds in distances, angles and torsion angles; correlations between esds in cell parameters are only used when they are defined by crystal symmetry. An approximate (isotropic) treatment of cell esds is used for estimating esds involving l.s. planes.
Refinement. Carbon and nitrogen-bound H atoms were placed in calculated positions and were included in the refinement in the riding model approximation, with *U*(H) set to 1.2 *U*_eq_(C) and *U*_eq_(N) respectively.The H atoms of the methyl groups were allowed to rotate with a fixed angle around the C-C bond to best fit the experimental electron density (HFIX 137 in the SHELX program suite (Sheldrick, 2008)), with U(H) set to 1.5Ueq(C).The H atoms of the hydroxyl groups were allowed to rotate with a fixed angle around the C—O bond to best fit the experimental electron density (HFIX 147 in the SHELX program suite (Sheldrick, 2008)), with *U*(H) set to 1.5*U*_eq_(C).

### Fractional atomic coordinates and isotropic or equivalent isotropic displacement parameters (Å^2^)

**Table:**

	*x*	*y*	*z*	*U*_iso_*/*U*_eq_	
O11	0.5156 (3)	1.2649 (3)	0.49062 (12)	0.0377 (7)	
H11A	0.5377	1.3476	0.4959	0.057*	
O12	0.6526 (3)	1.2222 (3)	0.55294 (12)	0.0349 (6)	
O13	0.8393 (3)	1.0813 (3)	0.48959 (12)	0.0340 (7)	
O14	0.8840 (3)	0.8724 (3)	0.51895 (13)	0.0363 (7)	
H14A	0.9655	0.8913	0.5107	0.054*	
O15	0.5589 (3)	0.5243 (3)	0.50160 (14)	0.0406 (8)	
O16	0.3406 (3)	0.5520 (3)	0.48128 (12)	0.0321 (6)	
O17	0.1613 (3)	0.7171 (3)	0.49938 (13)	0.0365 (7)	
H17A	0.2233	0.6602	0.4919	0.055*	
O18	0.1428 (3)	0.9376 (3)	0.51382 (13)	0.0351 (7)	
O21	0.5303 (3)	0.7790 (3)	0.38363 (14)	0.0370 (7)	
O22	0.5504 (3)	1.0018 (3)	0.37440 (17)	0.0500 (10)	
H22A	0.6137	1.0670	0.3758	0.075*	
O23	0.7235 (3)	1.1753 (3)	0.37790 (17)	0.0455 (9)	
O24	0.9469 (3)	1.1983 (2)	0.38543 (13)	0.0318 (6)	
O25	1.2733 (2)	0.8364 (3)	0.39696 (10)	0.0276 (6)	
H25A	1.3548	0.8232	0.3875	0.041*	
O26	1.2263 (3)	0.6532 (3)	0.35071 (11)	0.0291 (6)	
O27	1.0649 (3)	0.4957 (3)	0.41643 (10)	0.0265 (5)	
O28	0.8825 (3)	0.4524 (2)	0.36955 (11)	0.0286 (6)	
H28	0.9106	0.3703	0.3714	0.043*	
N3	0.9720 (4)	0.3343 (4)	0.49322 (15)	0.0420 (9)	
H3A	0.9185	0.2571	0.4910	0.050*	
H3B	1.0072	0.3520	0.4636	0.050*	
N4	0.3409 (3)	0.3907 (4)	0.39447 (13)	0.0310 (7)	
H4A	0.2799	0.4288	0.3734	0.037*	
H4B	0.3290	0.4338	0.4232	0.037*	
C10	0.5492 (3)	1.0341 (3)	0.51166 (12)	0.0190 (6)	
C11	0.6516 (3)	0.9326 (3)	0.50839 (12)	0.0180 (6)	
C12	0.6134 (3)	0.7951 (3)	0.50505 (13)	0.0203 (6)	
H12	0.6836	0.7263	0.5043	0.024*	
C13	0.4749 (3)	0.7538 (3)	0.50279 (12)	0.0181 (6)	
C14	0.3713 (3)	0.8560 (3)	0.50605 (12)	0.0182 (6)	
C15	0.4121 (3)	0.9940 (3)	0.51066 (13)	0.0193 (6)	
H15	0.3426	1.0631	0.5132	0.023*	
C16	0.5807 (3)	1.1841 (3)	0.52053 (13)	0.0214 (6)	
C17	0.8019 (3)	0.9708 (3)	0.50521 (13)	0.0207 (6)	
C18	0.4570 (4)	0.5988 (3)	0.49537 (14)	0.0238 (7)	
C19	0.2155 (3)	0.8358 (4)	0.50648 (14)	0.0239 (7)	
C20	0.7591 (3)	0.8638 (3)	0.38189 (13)	0.0186 (6)	
C21	0.8632 (3)	0.9671 (3)	0.38146 (13)	0.0185 (6)	
C22	1.0014 (3)	0.9250 (3)	0.38182 (13)	0.0200 (6)	
H22	1.0716	0.9938	0.3817	0.024*	
C23	1.0406 (3)	0.7872 (3)	0.38235 (12)	0.0158 (6)	
C24	0.9378 (3)	0.6852 (3)	0.38409 (12)	0.0173 (6)	
C25	0.7998 (3)	0.7253 (3)	0.38374 (13)	0.0194 (6)	
H25	0.7304	0.6558	0.3848	0.023*	
C26	0.6027 (3)	0.8836 (4)	0.38037 (15)	0.0249 (7)	
C27	0.8424 (4)	1.1233 (3)	0.38165 (15)	0.0266 (7)	
C28	1.1904 (3)	0.7498 (3)	0.37547 (13)	0.0201 (6)	
C29	0.9707 (3)	0.5344 (3)	0.39091 (12)	0.0186 (6)	
C31	1.0866 (6)	0.3067 (6)	0.5262 (2)	0.0530 (13)	
H31A	1.1372	0.2244	0.5154	0.080*	
H31B	1.0499	0.2906	0.5585	0.080*	
H31C	1.1492	0.3864	0.5267	0.080*	
C32	0.8850 (6)	0.4510 (7)	0.5079 (3)	0.0627 (16)	
H32A	0.9411	0.5354	0.5093	0.094*	
H32B	0.8457	0.4323	0.5397	0.094*	
H32C	0.8099	0.4633	0.4847	0.094*	
C41	0.3069 (5)	0.2421 (4)	0.40027 (17)	0.0353 (9)	
H41A	0.3220	0.1939	0.3698	0.053*	
H41B	0.2097	0.2326	0.4098	0.053*	
H41C	0.3664	0.2017	0.4250	0.053*	
C42	0.4831 (4)	0.4183 (5)	0.37732 (19)	0.0395 (10)	
H42A	0.4988	0.3687	0.3471	0.059*	
H42B	0.5498	0.3865	0.4014	0.059*	
H42C	0.4949	0.5179	0.3721	0.059*	

### Atomic displacement parameters (Å^2^)

**Table:**

	*U*^11^	*U*^22^	*U*^33^	*U*^12^	*U*^13^	*U*^23^
O11	0.0440 (17)	0.0134 (11)	0.0557 (19)	−0.0008 (11)	−0.0229 (14)	−0.0006 (12)
O12	0.0337 (14)	0.0235 (13)	0.0475 (17)	0.0014 (11)	−0.0156 (13)	−0.0105 (12)
O13	0.0244 (13)	0.0226 (12)	0.0550 (18)	−0.0073 (10)	0.0025 (12)	0.0038 (12)
O14	0.0114 (11)	0.0270 (13)	0.070 (2)	−0.0007 (10)	−0.0005 (12)	0.0107 (14)
O15	0.0245 (13)	0.0136 (11)	0.084 (3)	−0.0006 (10)	−0.0132 (15)	−0.0025 (13)
O16	0.0201 (12)	0.0194 (12)	0.0567 (18)	−0.0025 (9)	−0.0041 (12)	−0.0130 (12)
O17	0.0174 (12)	0.0261 (13)	0.066 (2)	−0.0019 (10)	0.0030 (13)	−0.0130 (13)
O18	0.0132 (11)	0.0260 (13)	0.066 (2)	0.0014 (10)	−0.0006 (12)	−0.0076 (13)
O21	0.0109 (11)	0.0223 (13)	0.078 (2)	−0.0005 (9)	−0.0032 (13)	0.0035 (14)
O22	0.0147 (12)	0.0226 (13)	0.113 (3)	0.0058 (10)	−0.0048 (16)	0.0069 (16)
O23	0.0191 (13)	0.0148 (12)	0.102 (3)	0.0052 (9)	−0.0021 (16)	−0.0011 (15)
O24	0.0222 (12)	0.0108 (10)	0.0624 (19)	−0.0022 (9)	−0.0031 (13)	0.0014 (11)
O25	0.0095 (10)	0.0280 (13)	0.0454 (16)	−0.0009 (9)	−0.0013 (10)	−0.0052 (11)
O26	0.0186 (11)	0.0230 (12)	0.0457 (16)	0.0041 (10)	0.0020 (11)	−0.0069 (11)
O27	0.0216 (12)	0.0175 (11)	0.0405 (15)	0.0036 (9)	−0.0048 (10)	0.0043 (10)
O28	0.0314 (13)	0.0099 (10)	0.0445 (16)	−0.0022 (9)	−0.0115 (11)	−0.0007 (10)
N3	0.051 (2)	0.0303 (17)	0.045 (2)	−0.0167 (16)	−0.0150 (18)	0.0110 (15)
N4	0.0235 (15)	0.0280 (16)	0.0414 (19)	0.0082 (12)	−0.0053 (13)	−0.0080 (14)
C10	0.0182 (14)	0.0144 (13)	0.0243 (16)	−0.0007 (11)	−0.0005 (12)	−0.0010 (11)
C11	0.0137 (13)	0.0149 (13)	0.0255 (16)	0.0009 (11)	−0.0005 (11)	0.0005 (11)
C12	0.0129 (14)	0.0152 (14)	0.0328 (18)	−0.0002 (11)	−0.0014 (12)	−0.0004 (12)
C13	0.0130 (13)	0.0119 (13)	0.0295 (17)	−0.0008 (11)	−0.0020 (12)	−0.0005 (11)
C14	0.0127 (13)	0.0161 (14)	0.0258 (16)	−0.0010 (11)	0.0010 (11)	−0.0023 (12)
C15	0.0124 (13)	0.0149 (14)	0.0305 (16)	0.0009 (10)	−0.0018 (12)	−0.0035 (12)
C16	0.0165 (14)	0.0143 (13)	0.0333 (18)	−0.0014 (11)	0.0003 (13)	−0.0035 (12)
C17	0.0147 (14)	0.0178 (14)	0.0297 (17)	−0.0020 (11)	0.0027 (12)	−0.0058 (12)
C18	0.0212 (15)	0.0131 (13)	0.0370 (19)	−0.0018 (12)	0.0005 (14)	0.0002 (13)
C19	0.0128 (14)	0.0244 (16)	0.0344 (19)	−0.0008 (12)	−0.0003 (13)	−0.0047 (14)
C20	0.0102 (12)	0.0152 (14)	0.0304 (17)	0.0013 (10)	−0.0002 (12)	−0.0016 (12)
C21	0.0153 (14)	0.0102 (12)	0.0299 (16)	0.0005 (10)	−0.0018 (12)	−0.0015 (12)
C22	0.0145 (13)	0.0118 (13)	0.0336 (17)	−0.0023 (10)	0.0007 (13)	−0.0016 (12)
C23	0.0092 (12)	0.0124 (13)	0.0258 (15)	−0.0009 (10)	−0.0019 (11)	−0.0008 (11)
C24	0.0134 (13)	0.0133 (13)	0.0253 (15)	−0.0013 (11)	0.0008 (12)	−0.0014 (12)
C25	0.0130 (13)	0.0139 (14)	0.0312 (17)	−0.0029 (11)	−0.0001 (12)	0.0007 (12)
C26	0.0106 (13)	0.0223 (15)	0.042 (2)	0.0011 (11)	−0.0007 (13)	−0.0013 (15)
C27	0.0236 (16)	0.0138 (14)	0.042 (2)	0.0024 (12)	−0.0014 (15)	−0.0009 (14)
C28	0.0112 (13)	0.0157 (14)	0.0334 (18)	0.0017 (10)	0.0010 (12)	0.0054 (12)
C29	0.0158 (14)	0.0135 (13)	0.0267 (16)	−0.0007 (11)	0.0006 (12)	0.0008 (11)
C31	0.051 (3)	0.052 (3)	0.056 (3)	0.004 (2)	−0.015 (2)	0.013 (2)
C32	0.043 (3)	0.076 (4)	0.069 (4)	0.015 (3)	0.019 (3)	0.024 (3)
C41	0.034 (2)	0.032 (2)	0.039 (2)	0.0016 (16)	−0.0071 (17)	−0.0011 (17)
C42	0.028 (2)	0.039 (2)	0.051 (3)	0.0064 (17)	0.0007 (18)	−0.001 (2)

### Geometric parameters (Å, º)

**Table:**

O11—C16	1.304 (5)	C11—C12	1.382 (4)
O11—H11A	0.8400	C11—C17	1.501 (4)
O12—C16	1.198 (5)	C12—C13	1.398 (4)
O13—C17	1.208 (4)	C12—H12	0.9500
O14—C17	1.297 (4)	C13—C14	1.409 (4)
O14—H14A	0.8400	C13—C18	1.522 (4)
O15—C18	1.232 (5)	C14—C15	1.396 (4)
O16—C18	1.274 (4)	C14—C19	1.518 (4)
O17—C19	1.276 (4)	C15—H15	0.9500
O17—H17A	0.8400	C20—C25	1.396 (4)
O18—C19	1.226 (4)	C20—C21	1.418 (4)
O21—C26	1.233 (4)	C20—C26	1.523 (4)
O22—C26	1.260 (4)	C21—C22	1.396 (4)
O22—H22A	0.8788	C21—C27	1.522 (4)
O23—C27	1.258 (4)	C22—C23	1.384 (4)
O24—C27	1.248 (4)	C22—H22	0.9500
O25—C28	1.304 (4)	C23—C24	1.400 (4)
O25—H25A	0.8400	C23—C28	1.504 (4)
O26—C28	1.211 (4)	C24—C25	1.388 (4)
O27—C29	1.214 (4)	C24—C29	1.504 (4)
O28—C29	1.307 (4)	C25—H25	0.9500
O28—H28	0.8400	C31—H31A	0.9800
N3—C31	1.463 (6)	C31—H31B	0.9800
N3—C32	1.465 (8)	C31—H31C	0.9800
N3—H3A	0.9100	C32—H32A	0.9800
N3—H3B	0.9100	C32—H32B	0.9800
N4—C42	1.479 (6)	C32—H32C	0.9800
N4—C41	1.481 (5)	C41—H41A	0.9800
N4—H4A	0.9100	C41—H41B	0.9800
N4—H4B	0.9100	C41—H41C	0.9800
C10—C15	1.381 (4)	C42—H42A	0.9800
C10—C11	1.396 (4)	C42—H42B	0.9800
C10—C16	1.501 (4)	C42—H42C	0.9800
			
C16—O11—H11A	109.5	C22—C21—C20	118.3 (3)
C17—O14—H14A	109.5	C22—C21—C27	114.6 (3)
C19—O17—H17A	109.5	C20—C21—C27	127.2 (3)
C26—O22—H22A	111.4	C23—C22—C21	122.9 (3)
C28—O25—H25A	109.5	C23—C22—H22	118.6
C29—O28—H28	109.5	C21—C22—H22	118.6
C31—N3—C32	113.5 (5)	C22—C23—C24	118.9 (3)
C31—N3—H3A	108.9	C22—C23—C28	119.6 (3)
C32—N3—H3A	108.9	C24—C23—C28	121.2 (3)
C31—N3—H3B	108.9	C25—C24—C23	119.0 (3)
C32—N3—H3B	108.9	C25—C24—C29	118.3 (3)
H3A—N3—H3B	107.7	C23—C24—C29	122.5 (3)
C42—N4—C41	114.6 (3)	C24—C25—C20	122.6 (3)
C42—N4—H4A	108.6	C24—C25—H25	118.7
C41—N4—H4A	108.6	C20—C25—H25	118.7
C42—N4—H4B	108.6	O21—C26—O22	121.7 (3)
C41—N4—H4B	108.6	O21—C26—C20	117.3 (3)
H4A—N4—H4B	107.6	O22—C26—C20	121.0 (3)
C15—C10—C11	118.7 (3)	O24—C27—O23	120.9 (3)
C15—C10—C16	118.0 (3)	O24—C27—C21	118.0 (3)
C11—C10—C16	123.1 (3)	O23—C27—C21	121.1 (3)
C12—C11—C10	119.4 (3)	O26—C28—O25	125.5 (3)
C12—C11—C17	119.4 (3)	O26—C28—C23	122.2 (3)
C10—C11—C17	121.1 (3)	O25—C28—C23	112.2 (3)
C11—C12—C13	122.2 (3)	O27—C29—O28	124.7 (3)
C11—C12—H12	118.9	O27—C29—C24	122.0 (3)
C13—C12—H12	118.9	O28—C29—C24	113.1 (3)
C12—C13—C14	118.5 (3)	N3—C31—H31A	109.5
C12—C13—C18	113.3 (3)	N3—C31—H31B	109.5
C14—C13—C18	128.2 (3)	H31A—C31—H31B	109.5
C15—C14—C13	118.3 (3)	N3—C31—H31C	109.5
C15—C14—C19	113.7 (3)	H31A—C31—H31C	109.5
C13—C14—C19	127.9 (3)	H31B—C31—H31C	109.5
C10—C15—C14	122.8 (3)	N3—C32—H32A	109.5
C10—C15—H15	118.6	N3—C32—H32B	109.5
C14—C15—H15	118.6	H32A—C32—H32B	109.5
O12—C16—O11	125.4 (3)	N3—C32—H32C	109.5
O12—C16—C10	122.5 (3)	H32A—C32—H32C	109.5
O11—C16—C10	112.0 (3)	H32B—C32—H32C	109.5
O13—C17—O14	124.8 (3)	N4—C41—H41A	109.5
O13—C17—C11	121.9 (3)	N4—C41—H41B	109.5
O14—C17—C11	113.2 (3)	H41A—C41—H41B	109.5
O15—C18—O16	122.8 (3)	N4—C41—H41C	109.5
O15—C18—C13	117.7 (3)	H41A—C41—H41C	109.5
O16—C18—C13	119.4 (3)	H41B—C41—H41C	109.5
O18—C19—O17	120.8 (3)	N4—C42—H42A	109.5
O18—C19—C14	117.8 (3)	N4—C42—H42B	109.5
O17—C19—C14	121.5 (3)	H42A—C42—H42B	109.5
C25—C20—C21	118.4 (3)	N4—C42—H42C	109.5
C25—C20—C26	113.6 (3)	H42A—C42—H42C	109.5
C21—C20—C26	128.0 (3)	H42B—C42—H42C	109.5
			
C15—C10—C11—C12	−1.0 (5)	C25—C20—C21—C22	1.4 (5)
C16—C10—C11—C12	173.4 (3)	C26—C20—C21—C22	−178.3 (3)
C15—C10—C11—C17	174.9 (3)	C25—C20—C21—C27	−177.2 (4)
C16—C10—C11—C17	−10.6 (5)	C26—C20—C21—C27	3.0 (6)
C10—C11—C12—C13	2.7 (5)	C20—C21—C22—C23	0.3 (5)
C17—C11—C12—C13	−173.3 (3)	C27—C21—C22—C23	179.1 (3)
C11—C12—C13—C14	−2.7 (5)	C21—C22—C23—C24	−2.0 (5)
C11—C12—C13—C18	175.6 (3)	C21—C22—C23—C28	171.1 (3)
C12—C13—C14—C15	0.9 (5)	C22—C23—C24—C25	2.0 (5)
C18—C13—C14—C15	−177.0 (3)	C28—C23—C24—C25	−171.1 (3)
C12—C13—C14—C19	−177.3 (3)	C22—C23—C24—C29	−172.2 (3)
C18—C13—C14—C19	4.7 (6)	C28—C23—C24—C29	14.7 (5)
C11—C10—C15—C14	−0.7 (5)	C23—C24—C25—C20	−0.2 (5)
C16—C10—C15—C14	−175.4 (3)	C29—C24—C25—C20	174.2 (3)
C13—C14—C15—C10	0.7 (5)	C21—C20—C25—C24	−1.5 (5)
C19—C14—C15—C10	179.2 (3)	C26—C20—C25—C24	178.3 (3)
C15—C10—C16—O12	123.0 (4)	C25—C20—C26—O21	4.0 (5)
C11—C10—C16—O12	−51.5 (5)	C21—C20—C26—O21	−176.3 (4)
C15—C10—C16—O11	−53.8 (4)	C25—C20—C26—O22	−173.7 (4)
C11—C10—C16—O11	131.6 (4)	C21—C20—C26—O22	6.1 (6)
C12—C11—C17—O13	150.8 (4)	C22—C21—C27—O24	−4.8 (5)
C10—C11—C17—O13	−25.2 (5)	C20—C21—C27—O24	173.9 (4)
C12—C11—C17—O14	−26.7 (5)	C22—C21—C27—O23	174.9 (4)
C10—C11—C17—O14	157.3 (3)	C20—C21—C27—O23	−6.4 (7)
C12—C13—C18—O15	14.0 (5)	C22—C23—C28—O26	−138.4 (4)
C14—C13—C18—O15	−168.0 (4)	C24—C23—C28—O26	34.7 (5)
C12—C13—C18—O16	−163.2 (4)	C22—C23—C28—O25	38.4 (5)
C14—C13—C18—O16	14.8 (6)	C24—C23—C28—O25	−148.6 (3)
C15—C14—C19—O18	−4.7 (5)	C25—C24—C29—O27	−138.4 (4)
C13—C14—C19—O18	173.7 (4)	C23—C24—C29—O27	35.8 (5)
C15—C14—C19—O17	175.2 (4)	C25—C24—C29—O28	37.1 (5)
C13—C14—C19—O17	−6.4 (6)	C23—C24—C29—O28	−148.6 (3)

### Hydrogen-bond geometry (Å, º)

**Table:**

*D*—H···*A*	*D*—H	H···*A*	*D*···*A*	*D*—H···*A*
N3—H3*A*···O13^i^	0.91	1.86	2.762 (4)	169
N3—H3*B*···O27	0.91	1.99	2.798 (5)	147
N4—H4*A*···O26^ii^	0.91	2.32	3.025 (4)	134
N4—H4*A*···O27^ii^	0.91	2.49	2.918 (4)	110
N4—H4*A*···O12^iii^	0.91	2.19	2.830 (5)	127
N4—H4*B*···O16	0.91	1.99	2.879 (5)	167
O11—H11*A*···O15^iv^	0.84	1.73	2.560 (4)	171
O14—H14*A*···O17^v^	0.84	2.55	3.119 (4)	126
O14—H14*A*···O18^v^	0.84	1.77	2.583 (4)	161
O17—H17*A*···O16	0.84	1.57	2.409 (4)	176
O22—H22*A*···O23	0.88	1.49	2.370 (4)	179
O25—H25*A*···O21^v^	0.84	1.75	2.572 (4)	164
O25—H25*A*···O22^v^	0.84	2.59	3.181 (4)	129
O28—H28···O24^i^	0.84	1.74	2.571 (3)	168
C32—H32*C*···O15	0.98	2.54	3.234 (6)	128
C41—H41*C*···O11^i^	0.98	2.41	3.235 (5)	142
C42—H42*C*···O21	0.98	2.57	3.519 (6)	164

Symmetry codes: (i) *x*, *y*−1, *z*; (ii) *x*−1, *y*, *z*; (iii) *y*−1, −*x*+1, *z*−1/4; (iv) *x*, *y*+1, *z*; (v) *x*+1, *y*, *z*.
